# Use of antenatal corticosteroids in Brazil: data analysis from the National Survey Nascer no Brasil

**DOI:** 10.1590/1984-0462/2022/40/2020126

**Published:** 2021-09-01

**Authors:** Antônio José Ledo Alves da Cunha, Karina Bilda de Castro Rezende, Maria Elisabeth Lopes Moreira, Silvana Granado Nogueira da Gama, Maria do Carmo Leal

**Affiliations:** a Universidade Federal do Rio de Janeiro, Rio de Janeiro, RJ, Brazil.; b Fundação Oswaldo Cruz, Instituto Fernandes Figueira, Rio de Janeiro, RJ, Brazil.

**Keywords:** Adrenal cortex hormones, Prenatal care, Obstetric labor, premature, Primary prevention, Risk factors, Corticosteroides, Cuidado pré-natal, Trabalho de parto prematuro, Prevenção primária, Fatores de risco

## Abstract

**Objective:**

To estimate the rate of the use of antenatal corticosteroids (ANC) among pregnant women and to identify the conditions associated with their non-use in Brazil.

**Methods:**

Secondary data analysis from “Birth in Brazil”, a national hospital-based survey carried out in 2011–2012 on childbirth and birth. The sample was characterized regarding maternal age, marital status and maternal education, parity, mode of delivery and place of residence. The association of ANC use with gestational age and type of delivery was analyzed. The studied maternal complications were the presence of hypertension, pre-eclampsia/eclampsia, and pyelonephritis, infection by the HIV virus or acquired immune deficiency syndrome.

**Results:**

2,623 pregnant women with less than 37 weeks of gestational age were identified, and, of these, 835 (31.8%) received ANC. The frequency of ANC use was higher among women with gestational ages between 26–34 weeks (481 cases; 48.73%). In pregnancies with less than 37 weeks, the use of ANC was 23.9% in spontaneous deliveries, 20.6% in induced deliveries and 43.8% among those who did not go into labor. The variables vaginal delivery (OR 2.5; 95%CI 1.8–3.4) and living in the countryside were associated with not using ANC, and the occurrence of pre-eclampsia/eclampsia (OR 1.8; 95%CI 1.2–2.9) was associated with the use of ANC.

**Conclusions:**

The use of ANC among Brazilian pregnant women was low. Interventions to increase its use are necessary and can contribute to reduce neonatal mortality and morbidity. ANC should be promoted in pregnancies of less than 37 weeks, especially in cases of vaginal delivery and for those living in the countryside.

## RESUMO

**Objetivo:**

Estimar a taxa de uso de corticosteroide antenatal (CAN) em gestantes e identificar condições associadas à não utilização do medicamento no Brasil.

**Métodos:**

Análise secundária de dados da Pesquisa Nascer no Brasil de 2011–2012, inquérito nacional de base hospitalar sobre parto e nascimento. A amostra foi caracterizada em relação a idade materna, estado civil e escolaridade da mãe, paridade, via de parto e local de moradia. Foram associados o uso de CAN e a idade gestacional do parto e o tipo do parto. As intercorrências maternas observadas foram a presença de hipertensão, pré-eclâmpsia/eclâmpsia, pielonefrite, infecção pelo vírus da imunodeficiência humana (HIV) ou síndrome de imunodeficiência adquirida.

**Resultados:**

Foram identificadas 2.623 gestantes com idade gestacional inferior a 37 semanas e, destas, 835 (31,8%) receberam CAN. A faixa de idade gestacional de 26–34 semanas foi a que mais recebeu, em 481 (48,7%) casos. Em gestação com menos de 37 semanas, a utilização de CAN em parto espontâneo foi de 23,9%; em parto induzido, de 20,6%; e naquelas que não entraram em trabalho de parto, de 43,8%. As variáveis parto vaginal (*Odds Ratio* — OR=2,5; intervalo de confiança de 95% — IC95% 1,8–3,4) e residir em cidades do interior estiveram associadas ao não uso do CAN. Ocorrência de pré-eclâmpsia/eclâmpsia (OR=1,8; IC95% 1,2–2,9) mostrou-se associada ao seu uso.

**Conclusões:**

O uso de CAN em gestantes brasileiras foi baixo. Intervenções para aumentar sua utilização são necessárias e podem contribuir para reduzir a mortalidade e a morbidade neonatal. Deve-se promover a utilização de CAN em gestações com menos de 37 semanas, especialmente em casos de parto vaginal e naquelas residindo em cidades do interior.

## INTRODUCTION

Premature birth is one of the main causes of neonatal mortality and morbidity. Even though the number of children who die before the age of 5 has reached a new minimum — 5.6 million in 2016, in comparison to almost 9.9 million in 2000 —, the proportion of these deaths in the neonatal period raised from 41 to 46% during the same period, globally.^1^ In countries of Sub-Saharan Africa and the South of Asia, the death rates of newborns (NB) is not decreasing fast, especially regarding children aged from 1 to 5 years. As a result, newborns respond for the growing proportion of children dying every year.^2^

The administration of corticosteroids before birth in premature births is an efficient prenatal therapy available to induce fetal maturation and decrease unfavorable outcomes for the newborn.^3^ ^,^ ^4^ Besides, it is the most efficient intervention to reduce preterm neonatal mortality, when administered to pregnant women at risk of premature birth.^4^ ^,^ ^5^

Considering the importance of premature NB mortality and the efficacy of the use of antenatal corticosteroids (ANC), there are indications that the global absorption of this intervention has been low.^6^ ^,^ ^7^ Some authors justify that this is partly owed to the limited studies that examine the possible harmful effects of ANC in environments with scarce resources.^8^ Despite the little knowledge about the use of ANC in health units in low and middle-income countries, the Brazilian Neonatal Research Network (RBPN) verified that the use of ANC in its centers, represented by university hospitals, was lower than 70% in 2011, in pregnancies between 23 and 33 weeks.^9^

The objectives of this study were to estimate the rate of use of ANC among pregnant women and to identify the conditions associated with the non-use of the medication in Brazil.^9^

## METHOD

This study used the data base from Nascer no Brasil, a Brazilian hospital-based study composed of puerperal women and their newborn children, carried out between 2011 and 2012, in public, private and mixed hospitals — private hospitals associated with the Brazilian National Health System (SUS).

The study sample was constituted of women and their children, and defined in three stages. The first stage included hospitals with more than 500 births/year, stratified according to the country’s regions (North, Northeast, South, Southeast, Midwest). In the second stage, using the reverse sampling method, researchers defined the number of necessary days to interview 90 puerperal women in each one of the 226 selected hospitals (minimum of seven days). In the third stage, the puerperal women and their newborns were selected. The women were sampled with equal probability between those who were eligible and were admitted to the hospital on that day. The eligible women were the ones who gave birth to a living fetus or a stillborn in the selected maternity wards until we completed the sample of 90 puerperal women. Those with difficulties to communicate, both for severe mental illness or for not speaking Portuguese, were excluded.

A group of interviewers trained by the central coordination conducted face to face interviews with the puerperal women in the first 24 hours after birth during hospital stay, and extracted data from their charts and the newborns’. Information was also collected after hospital discharge (or death) through electronic forms. In case of prolonged hospital stay, the data were collected 42 days after hospitalization and after birth for women; and for newborns, after the 28^th^ day (neonatal period). In case of hospital transfer for the woman and/or the newborn, the data were collected from the hospital, even when it was not part of the sample of the selected health institutions.

Refusals or early discharge were replaced by a new selection of puerperal women in the same hospital. Prenatal cards, when available, were photographed and, afterwards, the relevant data were extracted and scanned in electronic forms. Telephone follow-up interviews were conducted before six months and 12 months after birth, to obtain information about maternal and neonatal outcomes. All of the field work was carried out by professionals or students in the health field, with the supervision of the research group. Additional data about the sample design can be found in the study of reference.^10^ ^,^ ^11^

In every statistical analysis, we considered the complex sampling design. Data weighting was calculated by the inverse probability of inclusion of each puerperal woman in the sample. To make sure that the distribution of interviewed puerperal women was similar to that observed in the births of the population sampled in 2011, a calibration procedure was used in each selection stratum.^10^ The whole analysis was carried out using the Statistical Package for the Social Sciences (SPSS) software, version 17, (SPSS Inc., Chicago, United States).

The use of corticoids is the dependent variable (yes or no), and the data was obtained by the chart of the pregnant woman and the answers to the interviews. The use of the medication was associated with gestational age at birth, stratified in:

- Up to 25 weeks;
- 26 to 33 weeks and 6 days.
- 34 to 36 weeks and 6 days.
- 37 weeks or more.

Such a use was also stratified according to type of birth, classified as:

- Spontaneous.
- Induced.
- Did not go into labor.

The sample was characterized regarding:

- Maternal age, stratified from 12 to 19 years, 20 to 34 years, and 35 years or older.
- Maternal marital status, classified as married or not married.
- Maternal schooling, grouped in incomplete elementary school (ES), complete ES, high school or higher education.
- Parity, grouped in primiparous, one or two previous births, or three or more previous births.
- Type of childbirth, vaginal or C-section.
- Place of household, capital or countryside.

The observed maternal intercurrences were the presence of arterial hypertension, pre-eclampsia/eclampsia, pyelonephritis, infection by the human immunodeficiency virus (HIV) or acquired immunodeficiency syndrome. These characteristics were considered as the independent variables for the logistic regression to evaluate the use of ANC. Variables without information were excluded from the tables. The study of association only contemplated premature newborns (<37 gestational weeks).

This study was approved by the Research Ethics Committee of Escola Nacional de Saúde Pública Sergio Arouca, in Fundação Oswaldo Cruz (ENSP/Fiocruz), report n. 92/2010. All of the procedures were adopted in order to guarantee the confidentiality of the information. Before each interview, the woman provided a written informed consent form.

## RESULTS

The study assessed 23,960 pregnant women. We excluded 142 (0.6%) cases that did not have complete information, and the final sample resulted in 23,818 cases. Among them, 895 reported having taken ANC (3.8%). Of the analyzed total, 2,623 pregnant women presented gestational age of less than 37 weeks. Of these puerperal women, 835 received ANC (31.8%). Among the pregnant women whose gestational age was less than 25 weeks, 18 (35.3%) took ANC. The same was true for 482 (48.7%) pregnant women in the same group of 26-34 weeks, and 336 (21.2%) in the group of 35-37 weeks.

Regarding type of childbirth, it was spontaneous in 1,219 cases, and induced in 481 cases. The use of ANC before 37 weeks was observed, respectively, in 291 (23.9%) and in 63 (20.6%) cases. For those who did not go into labor, the use of the medication was 482 out of 1,098 cases (42.8%) (Table 1).

**Table 1:** Use of antenatal corticosteroid per type of birth, according to gestational age.

	Did not receive corticosteroid				Received cortocosteroid			
	Spontaneous CB	Induced CB	No CB	Total	Spontaneous CB	Induced CB	No CB	Total
Until 25 weeks	21 63.6%	2 6%	10 30.4%	33	15 83.3%	0	3 16.7%	18
26 to 33+6 weeks	280 55.3%	47 9.3%	179 35.4%	506	177 35.3%	24 5%	280 58.2%	481
34 to 36+6 weeks	627 50.2%	194 15.5%	428 34.3%	1,249	99 29.5%	39 11.6%	198 58.9%	336
37 weeks or more	10,164 48.1%	3,870 18.3%	7,101 33.6%	21,135	28 46.7%	7 11.7%	25 41.6%	60

CB: child birth; n=142 (spontaneous CB=65; induced CB=17; did not go into CB=60).

In pregnancies of less than 37 weeks, the variables vaginal birth, in comparison to C-section (*Odds Ratio* – OR=2.5; 95% confidence interval – 95%CI 1.8–3.4) and living in the countryside, in comparison to living in the capitals (OR = 2.1; 95%CI 1.3–3.3), were associated with the non-use of ANC during pregnancy. In cases of pre-eclampsia or eclampsia (OR=1.8; 95%CI 1.2–2.9), the association was positive for the use of the medication (Table 2).

**Table 2:** Variables associated with the non-use of antenatal corticosteroids. Brazil, 2012.

Exposure		Crude *Odds Ratio*	95%CI		Adjusted *Odds Ratio* *	95%CI	
			Inferior limit	Superior limit		Inferior limit	Superior limit
Maternal age (years)							
	From 12 to 19 *vs*. 35 or more	1.04	0.60	1.80	1.17	0.59	2.33
	from 20 to 34 *vs*. 35 or more	0.59	0.26	1.33	0.96	0.35	2.66
Maternal marital status							
	Married *vs*. Not married	0.51	0.35	0.75	0.82	0.51	1.32
	Maternal schooling						
	Incomplete elementary school *vs*. Complete higher education and more	0.32	0.16	0.66	0.60	0.26	1.39
	Complete elementary school *vs*. Complete higher education and more	0.29	0.14	0.58	0.50	0.21	1.19
	Complete high school *vs*. Complete higher education and more	0.77	0.37	1.58	0.94	0.40	2.19
Parity							
	Primiparous *vs*. Three or more previous births	1.87	0.90	3.86	1.97	0.85	4.56
	One/two *vs*. Three or more previous births	1.18	0.63	2.21	1.32	0.69	2.53
Type of birth							
	Vaginal *vs*. C-section	2.85	2.12	3.84	**2.50**	**1.78**	**3.44**
Hypertension							
	No *vs*. yes	1.68	0.70	4.06	0.73	0.27	1.93
Pre-eclampsia/eclampsia							
	No *vs*. yes	3.09	1.85	5.15	**1.85**	**1.17**	**2.92**
Pyelonephritis							
	No *vs*. yes	1.79	0.65	4.89	1.27	0.36	4.46
HIV/Aids							
	No *vs*. yes	0.92	0.16	5.26	1.06	0.14	7.88
Place of household							
	Countryside *vs*. capital	1.96	1.20	3.12	**2.08**	**1.28**	**3.33**

95%CI: 95% confidence interval

* adjusted for all of the exposure variables in the; HIV: human immunodeficiency virus.

## DISCUSSION

This study estimated the rate of use of ANC among pregnant women, in a secondary data analysis of the study Nascer no Brasil, besides identifying the conditions associated with the non-use of the medication in Brazil. Two thousand, six hundred and twenty three pregnant women (31.8%) took ANC with gestational age of less than 37 weeks. The gestational age that most received ANC was 26-34 weeks. The use of ANC was more frequent among the pregnant women who did not go into labor (43.8%). In those with spontaneous birth, it was 23.9% and, in induced labors, 20.6%. The variables vaginal birth (vs. C-section) and living in the countryside (vs. capitals) were independently associated to the non-use of ANC. The occurrence of pre-eclampsia or eclampsia was positively associated to the use of ANC.

Since 1972,^12^ the beneficial effects of ANC have been repeatedly shown in pregnancies with risk of premature birth before 34 gestational weeks. There are no controversies about the fact that women with premature birth, of less than 34 weeks, should be treated with ANC. In any clinical condition, such as premature rupture of membranes, multiple pregnancies, pre-eclampsia and fetal growth restriction, the ANC is indicated,^13^ since it reduces neonatal mortality and morbidity; however, there are few studies reporting the rates of use of this conduct, especially in developing countries, where the existing analyses present inconsistent results.^14^

In 2008,^15^ the use of ANC before preterm birth was assessed in nine hospitals of four countries in the Southeast of Asia, including Indonesia, Malaysia, the Philippines and Thailand, through the revision of medical records of 9,550 women. The administration of corticosteroids to women who gave birth before 34 gestational weeks ranged widely between these countries (9 to 73%) and among the hospitals in each country (0-86%), similarly to this study. The authors concluded that the assessment of potential facilitating factors or barriers for the acceptance of this effective antenatal intervention in hospitals is necessary, so that the use of ANC be uniform and homogeneous.

In Gana,^16^ in 2018, it was demonstrated that two thirds of 93 children born with less than 34 gestational weeks, hospitalized at an intensive care unit (ICU), received ANC. The authors concluded that, in order to improve the survival and morbidity of the preterm, it was urgent and necessary to increase the use of corticosteroid before the premature deliveries in Gana and in other low and middle-income countries. However, the reduced number of analyzed cases limits any generalization.^16^ This inter-hospital variation was also observed in the hospitals of the Vermont Oxfort Network,^17^ which includes a collaborative volunteer group that created and maintains a database of very low-weight newborns assisted in their institutions.

A study from Australia and New Zealand^18^ reported that adherence to the current protocol regarding the recommendation as to the use of ANC was high in pregnancies of <35 weeks, and pointed out to the irregular use of ANC in situations that were not established by the local protocol. The authors concluded that the adherence to the recommendations of the protocol would reduce the exposure of ANC to infants for whom there is no evidence of benefit.

In Barcelona, a retrospective study with 1,083 premature infants showed that 42% received ANC. In those with less than 34 gestational weeks, there was reduction in the risk of death without changes in morbidity.^19^ Even if the methodologies and the design of the several presented studies^15^ ^–^ ^19^ have been different, it is possible to observe a wide variety regarding the use of ANC in comparison to studies from several countries.

Our findings are not very different from the ones found in the literature. RBPN described, in 2004,^20^ that more than 60% of the pregnant women who went into labor with less than 35 gestational weeks used ANC; however, only hospitals in RBNP were considered, which are neonatal units of university or research hospitals, where it is expected to be more knowledge and adherence to good practices and recommendations. Another study in RBPN, in 2010,^21^ verified that the use of ANC has ranged between 12 and 88% in eight public, tertiary and university maternity wards. In our sample, which is national, including general hospitals, we observed that in pregnancies in which the ANC would be recommended, it was used in less than half of the cases. This finding demonstrates there is much room to improve the use of this technology, which is acknowledged as an important and effective therapy for the improvement of neonatal outcomes.

As to the low use of ANC in pregnancies with gestational age of less than 37 weeks, our results are in accordance with recent evidence about the benefit of ANC in the late pre-term period^13^ ^,^ ^22^ ^,^ ^23^, and even justify its incorporation in clinical guidelines.^24^ However, this evidence became clear in 2016 and 2017, and our data refer to the biennial 2011/2012, when such a strategy was not universal.

Therefore, we observe that the potential to develop the use of ANC is higher in pregnancies that result in late pre-term labors, and only one fifth received the medication. It is worth to mention that the use of ANC in gestational ages of more than or equal to 37 weeks, situation when it is not recommended, was very unusual (0.28%), unlike the results observed in Australia and New Zealand.^18^ This suggests that the age group in which the use of ANC is not indicated should be known. However, in pregnancies for which its use is indicated and recommended, other factors make it so that it is not used.

Regarding the factors associated with the non-use of ANC, when it should have been indicated, we observed that the countryside cities of the Brazilian states showed less frequent use in comparison to capitals. This may have been owed to the knowledge and access to new technologies in the capitals. It is also possible that health professionals be more updated than those in the countryside.

We could observe the more frequent use of ANC in cases of pregnancies that resulted in vaginal birth. Pregnancies with operative birth include selective and planned C-sections, and may receive more attention from health professionals; so, the use of ANC can be more recommended. Besides, these cases contemplate the diseases that come with or are a result of pregnancy, whose guidelines may recommend the use of ANC in prematurity. Pregnancies with pre-eclampsia or eclampsia received more ANC. Birth is the only definitive treatment for pre-eclampsia and eclampsia, and its anticipation is often necessary to prevent more unfavorable outcomes for the mother; so, pre-eclampsia/eclampsia is the main cause of premature birth due to medical indication in the world.^25^ ^,^ ^26^ The administration of ANC is part of the protocol in cases of severe pre-eclampsia with gestational age of less than 34 weeks, with interruption of the pregnancy after 48 hours, when the clinical and laboratory response is favorable.^27^

Our study has some limitations to be considered. The data we used were obtained through medical charts, which may have caused verification errors, besides the memory bias resulting from maternal response. However, we believe that these errors, if they occurred, were probably in the sense of not registering the use of corticosteroids, thus not reducing the importance of our findings. This is a secondary analysis, which allowed us to evaluate the information in the database of the study Nascer no Brasil. We did not verify the used dose nor did we weight the number of times in which ANC was administered, which does not interfere in the evaluation of the rate of prescription nor its use. The data from 2011-2012 are not contemporary, but represent a national scenario. It is important to mention that, in this period, the recommendation as to the administration of ANC for cases of risk of premature birth after 34 weeks was already acknowledged, and its use was not recommended after this gestational period.^4^ We do not know if the data have changed throughout the years. A new edition of the study Nascer no Brasil has been approved, whose data will be compared to the ones presented here and will serve as an indicator of the quality and evolution of care provided in the country.

The little use of ANC documented in this study reflects flaws in adherence to clinical protocols, and emphasizes the need for educational actions about the importance of using the medication in pregnancies at risk for premature birth, as recommended by the World Health Organization.^28^ The verification that 21.2% of the analyzed sample used ANC in cases between 34 and 37 weeks may represent a flaw in care, since the recommendations as to the use in late prematurity were only published in 2016.^22^ ^,^ ^23^ Despite these limitations, our findings justify the prioritization of strategies to increase the use of ANC in Brazil.

To sum up, we verified that the use of ANC among the analyzed pregnant women with less than 37 gestational weeks was reduced. Interventions to increase its use are necessary and can contribute with the reduction of neonatal mortality and morbidity. Therefore, there must be a promotion of its use in pregnancies of less than 37 weeks, especially in cases of vaginal birth and those living in countryside cities. Even in remote health units, it is now possible to access institutional clinical protocols, which are available through digital means. Considering the efficacy, cost and availability of this intervention, it should be incorporated in institutional clinical protocols that are periodically updated; the access to them must be enabled and encouraged. Such measures have the potential to contribute with the reduction of child mortality.
